# Four year experience of sarcoma of soft tissues and bones in a tertiary care hospital and review of literature

**DOI:** 10.1186/1477-7819-9-51

**Published:** 2011-05-17

**Authors:** Tayyaba Z Ansari, Nehal Masood, Asra Parekh, Rabab Z Jafri, Syed N Niamatullah, Adnan A Zaidi, Masood Umer

**Affiliations:** 1 Aga Khan University Hospital, Stadium Road, P.O. Box 3500, Karachi 74800, Pakistan; 2 Liaquat National Hospital, National Stadium Road, P.O. Box 3500, Karachi 74800, Pakistan; 3 Shaukat Khanum Cancer Memorial Hospital, Main Clifton Road, Clifton, Karachi, Pakistan

## Abstract

**Background:**

Sarcoma encompasses an uncommon group of cancer and the data is insufficient from Pakistan. We report our four years experience of Sarcoma of soft tissues and bones.

**Methods:**

This cross sectional study was carried out at Aga Khan University Hospital from 2004 to 2008. The patients were divided into two groups from the outset i.e. initially diagnosed and relapsed group and separate sub group analysis was conducted.

**Results:**

Out of 93 newly diagnosed patients, 58 belonged to bone sarcoma and 35 to soft tissue sarcoma group. While for relapsed patients, 5 had soft tissue sarcoma and 9 had bone sarcoma. Mean age was 32.5 years. At presentation, approximately two third patients had localised disease while remaining one third had metastatic disease. The Kaplan Meier estimate of median recurrence free survival was 25 months, 35 months, and 44 months for Osteogenic sarcoma, Ewing's sarcoma and Chondrosarcoma respectively. For Leiomyosarcoma and Synovial sarcoma, it was 20 and 19 months respectively. The grade of the tumour (p = 0.02) and surgical margin status (p = 0.001) were statistically significant for determination of relapse of disease.

**Conclusion:**

The median recurrence free survival of patients in our study was comparable to the reported literature but with significant lost to follow rate. Further large-scale, multi centre studies are needed to have a more comprehensive understanding of this heterogeneous disease in our population.

## Background

The skeleton and soft tissue comprise approximately 75% of the average body weight but the cancer arising from these parts represent 1% of adult and 15% of pediatric malignancies [1]. The diversity and rarity of occurrence make their comprehensive understanding a difficult task. Broadly, sarcoma comprise of two distinct entities i.e. Bone sarcoma and Soft tissue sarcoma. Sarcomas have been associated with earlier radiation therapy, toxic exposure and genetic conditions but no clearly defined aetiology has been identified [2]. The histological grade of the tumour is one of the most important prognostic variables for soft tissue sarcoma [1,3,4]. Complete staging and treatment planning by multidisciplinary team of cancer specialists is required to determine the optimal treatment for these patients [5]. The role of chemotherapy is less well defined for soft tissue sarcoma while some bone sarcomas are chemo-sensitive and therefore, chemotherapy is an integral component of their treatment protocols. Bone sarcomas disseminate almost exclusively through the blood as bones lack a lymphatic system. Early lymphatic spread to regional nodes has only rarely been reported [2]. It usually affects the younger age group, and hence the social burden of disability and morbidity is huge for any community.

With this background, we planned to under take a study to report our four year experience of dealing with sarcoma patients at a tertiary care hospital as data is limited from our country. Our population exhibits a very diverse behaviour in terms of tumour biology, disease manifestation and outcome and we wanted to explore these factors in our ethnically varied population.

## Materials and methods

This descriptive, cross sectional study was conducted at Aga Khan University Hospital, Karachi. Our centre gets referral from all over the country. The method of referral is, however, not uniform. Sometimes primary physicians refer the patients and in other instances people make self-referrals.

The charts of the patients with diagnosis of the sarcoma were reviewed from a period of 2004 to 2008 after formal approval from the Institutional Ethical Review Board. Only adult patients with age of more than 16 years were included.

## Statistics

The data was analyzed in SPSS version 16.0. The descriptive analysis was done for the demographics and clinical characteristics of the patients. From the outset, two separate descriptive analyses were done, for relapsed and no relapsed cases. The further analysis was done on the basis of diagnosis whether soft tissue or bone sarcoma, localised versus metastatic disease and different modalities of treatment. The Chi square test was applied for univariate analysis for determining the significance of individual categorical variables for recurrence and Kaplan Meier survival curves were obtained for recurrence free survival.

## Results

**Figure 1:**
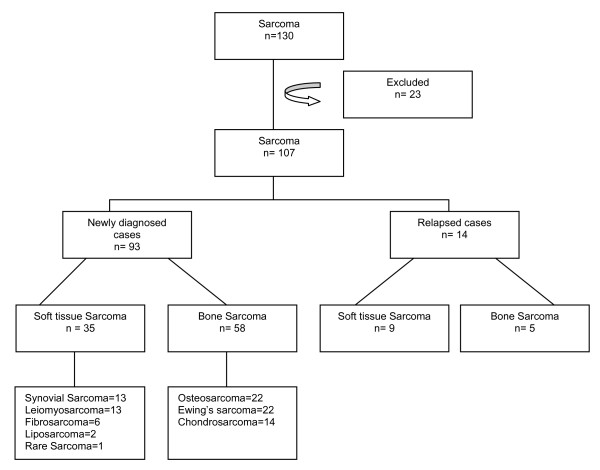
**Schematic Representation of Distribution of Entire Study Cohort Patients**. Out of 107 analyzed cases, 93 were newly diagnosed and 14 were relapsed cases. Among newly diagnosed patients, 35 patients had soft tissue sarcoma and 58 had bone sarcoma. Synovial Sarcoma, Leiomyosarcoma, Fibrosarcoma and Liposarcoma were the main subtypes of soft tissue sarcoma. While for bone sarcoma, Osteosarcoma, Ewing's sarcoma and Chondrosarcoma were the main diagnosis.

The total number of patients with the diagnosis of Sarcoma was found to be 130 during this four year period but the analysis was done for only 107 patients as 23 patients were excluded either because of inability to retrieve the charts from the Medical Record department or inadequate information in the charts. The analysis, thus done, comprised of 14 relapsed patients who had been referred for relapsed disease after having received the initial treatment somewhere else and 93 newly diagnosed patients. The distribution of patients is shown in the flow diagram Figure 1.

**Figure 2:**
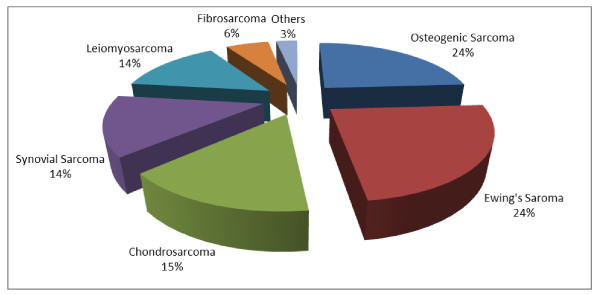
**Percentage of Sub Categories of Sarcoma**. Among 93 newly diagnosed patients, the percentage of Osteosarcoma, Ewing's sarcoma, Chondrosarcoma, Synovial sarcoma, Leiomyosarcoma, Fibrosarcoma and other rare sarcoma were 24%, 24%, 15%, 14%, 14%,6% and 3% respectively.

Among newly diagnosed patients in bone sarcoma category, 22 patients (23.7%) had Osteogenic sarcoma, 22 patients (23.7%) had Ewing's sarcoma and 14 patients (15%) had Chondrosarcoma. The soft tissue sarcomas were found to be less frequent in our analysis. The soft tissue sarcomas included Synovial sarcoma, Leiomyosarcoma, Fibrosarcoma, Liposarcoma and other rare sarcomas. The Synovial sarcoma and Leiomyosarcoma were found in 13 patients (14%) each, followed by Fibrosarcoma 6 patients (6%), Liposarcoma in 2 patients (2%) and the other rare sarcomas in less than 1% of the cases. (Figure 2 Pie chart)

The mean age group for the whole study population was 32.5 years with the range of 18 to 47 years. The stage of the disease at presentation was generally similar in both group of sarcoma patients with 67.8% of patients had localised disease and 34% had metastatic disease while staging details were missing for 2 patients with Ewing's sarcoma. The median follow up duration was 44 months (18-72 months).

### Osteogenic Sarcoma

There was unequal gender distribution in Osteogenic sarcoma with the male to female ration of 2.8:1. The diagnosis had been established by needle (trucut) biopsy for most of cases, but a few cases had excisional biopsies and exploratory surgeries as part of investigative work up.

The sub-group analysis of 22 patients with Osteogenic sarcoma showed that 16 had localised disease and 6 had metastatic disease at presentation. The long bones of the extremity were the main site of involvement in Osteogenic sarcoma with 17 cases (77%) of lower extremity and 4 cases (18%) of upper extremity location, while the site was not documented for one patient. Of these 16 patients with localised disease, twelve received neoadjuvant chemotherapy. Most patients tolerated the chemotherapy well and the commonly encountered side effects were nausea, vomiting, and mucositis. Only few patients had febrile neutropenia (grade 4 toxicity) but recovered fairly quickly with no definite localizing source of fever. Seven patients in the neo-adjuvant group had a good clinical response to the therapy while 2 had confirmed responsive disease radiologically as per RECIST criteria; however, the re-staging work up was missing for 3 patients.

**Figure 3:**
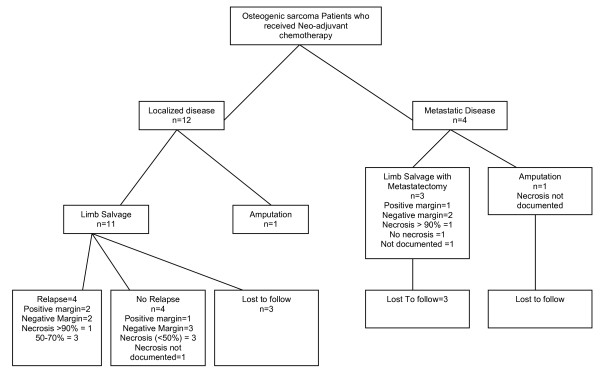
**Distribution of Osteosarcoma Patients who Received Neo-adjuvant Chemotherapy**. 16 patients with osteosarcoma received neoadjuvant chemotherapy. This included 4 patients with metastatic disease and 12 patients with localised disease. 11 of these 12 patients underwent limb salvage surgery but one ended up having amputation. 3 patients were lost to follow after limb salvage surgery while 4 relapsed and 4 did not relapse as per last follow up. The flow chart also shows the postoperative pathological characteristics of the relapse and no relapse patients.

The neoadjuvant chemotherapy was also given to 4 patients with metastatic lung nodules and subsequently 3 of them had limb salvage surgery with metastatectomy of lung nodules in the same sitting. Nevertheless, 1 patient did not have good response to neoadjuvant chemotherapy and underwent amputation. The histopathology revealed a variable percentage of necrosis; more than 90 percent in two patients, 50 to 70 percent in three, and less than fifty percent in three patients. Nonetheless, one patient did not have any necrosis after neo-adjuvant chemotherapy and there had been no comment on the percentage of necrosis in the final histopathology report for three patients. Among the four patients in the metastatic group who received neoadjuvant chemotherapy, one had more than 90 percent necrosis, 1 patient did not have any necrosis, while the percentage of necrosis has not been documented for the other two. The details are shown in Figure 3.

The two categorical variables significantly associated with the relapse of the disease in univariate analysis were grade of the tumour (p = 0.02) and surgical margin status (p = 0.001) as shown in Table 1.

**Table 1:** Univariate Analysis of Categorical Variables for Relapse of the Disease

	Number of Patients n (%)		
Variables	Relapse	No relapse	***p *****value**
**Grade of the Tumour**			
High grade	39(41.9%)	25(26.9%)	**0.02**
Low grade	0(0%)	4(4.3%)	
Not Documented^1^	0(0%)	3(3.2%)	

**Positive Surgical Margins**			
Yes	5(5.4%)	4(4.3%)	**0.001**
No	15(16.1%)	26(28%)	
Not Documented^2^	19(20.4%)	2(2.2%)	

^1 ^Grade of Tumour was not documented in 5 patients and outcome information was missing in 22.

^2 ^Margin status was not documented in 31 patients and outcome information missing for 10 patients

Grade of the tumour was classified pathologically as high or low grade. The margin was considered positive if tumour was present within 1 mm of resected specimens. The *p*-values were statistically significant for the grade of the tumour and the margins status post-operatively in determining the relapse of the disease.

**Figure 4:**
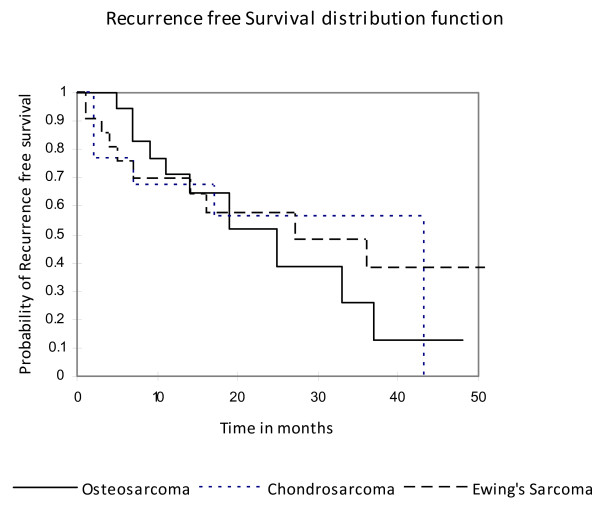
**Recurrence-free Survival of Bone Sarcoma**. The time in months is shown along x- axis and probability of survival along y-axis. The solid line indicates the recurrence free survival of Osteosarcoma, the dotted lines indicates for Chondrosarcoma and dashed lines depicts for Ewing's sarcoma. 3 year recurrence free survival was 25% for Osteosarcoma, 57% for Chondrosarcoma, and 49% for Ewing's sarcoma.

The relapse rate after limb salvage and amputation could not be compared directly because of small number of patients who underwent amputation. The patients who relapsed among limb salvage surgery versus no relapse in same group could also not be compared for the same reason of having fewer numbers of patients. However, the recurrence free survival for three different sub-types of bone sarcoma is shown in Figure 4. The Kaplan Meier estimate of median recurrence free survival was 25 months, 35 months, and 44 months for Osteogenic sarcoma, Ewing's sarcoma and Chondrosarcoma respectively. Four patients with Osteogenic sarcoma had recurrent disease at primary site while rest presented with distant recurrence predominantly with lung nodules detected on routine surveillance imaging.

### Ewing's Sarcoma

The gender distribution was 1:1 in Ewing's sarcoma. The neoadjuvant chemotherapy was given to 14 patients with Ewing's sarcoma and among them 3 had metastatic disease. The most commonly used chemotherapy regimen comprised of Cyclophosphamide, Vincristine, Adriamycin, Ifosfamide and Etoposide. The local treatment for localised disease was addressed at 12 - 14 weeks depending on the response to chemotherapy [6].

### Chondrosarcoma

Patients with localised Chondrosarcoma had primary upfront surgery. Out of 14 patients 11 had localised disease and only one patient had positive margins postoperative and treated by adjuvant radiation.

### Soft Tissue Sarcoma

The soft tissue sarcoma was under represented in our study and showed a trend towards female preponderance.

Majority of the patients i.e. 29 patients (31.2%) with soft tissue sarcoma had upfront surgery with negative margins in 21 patients and positive in 3 patients while margin status was not documented for 10 patients. Most patients had high grade tumour and five patients with Leiomyosarcoma received anthracyclines and Ifosfamide based adjuvant chemotherapy.

**Figure 5:**
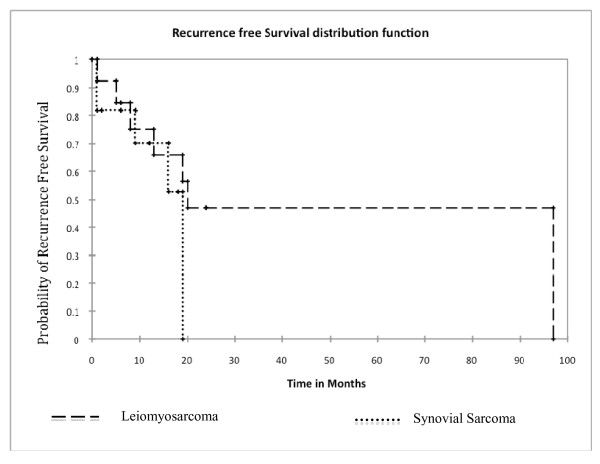
**Recurrence-free Survival of Two Main Types of Soft Tissue Sarcoma**. The probability of survival along y- axis is plotted as function of time in months along x-axis for 2 major sub-types of soft tissue sarcoma. The dotted line indicates Synovial sarcoma and dashed represents Leiomyosarcoma. The median survival was only 19 months for Synovial sarcoma and 20 months for Leiomyosarcoma.

The recurrence free survival is shown in Figure 5. The recurrence free survival for Leiomyosarcoma and Synovial sarcoma was 20 and 19 months respectively.

The outcome of entire cohort of newly diagnosed cases has been shown in Table 2.

**Table 2:** Outcome of Entire Cohort of Newly Diagnosed Sarcoma Patients

Outcome	Osteo-sarcoma	Chondro-sarcoma	Ewing's Sarcoma	Lipo-sarcoma	Fibro-sarcoma	Synovial Sarcoma	Leiomyo-sarcoma	Total
Alive with no recurrence	8	4	7	2	1	3	3	29

Alive with recurrence and taken treatment	0	0	0	0	0	2	0	2

Alive with recurrence/on supportive care	0	1	1	0	0	3	1	6

Died	1	2	4	0	3	0	3	13

Lost to follow	13	7	10	0	2	5	6	43

Total	22	14	22	2	6	13	13	93

The outcome of all the newly diagnosed patients is categorized on the basis of whether they are alive without recurrence, with recurrence but on treatment or supportive care, died or lost to follow.

## Discussion

Sarcomas represent the heterogeneous group of cancer with diverse tumour biology. Chemotherapy, being the main stay of treatment for certain sub-types of bone sarcoma e.g. Ewing's sarcoma, has proved to improve the recurrence free survival in adjuvant setting in Osteogenic sarcoma [7], but has a controversial role in soft tissue sarcomas. Wide adequate surgical resection with pathologically proven clear margins is the most effective therapeutic approach for management of soft tissue sarcoma.

Though the numbers of patients were small in our study but this was consistent with the overall incidence of the sarcoma and the rarity of the disease [8]. The age group affected comprised of young adults with predominance of males in Osteogenic sarcoma as compared to females which was in contrast to the reported literature [2]. It is not known whether this gender difference is related to epigenetic factors in our population or reflects a general social background of the community where men are more privileged than women and hence the greater access to medical facilities. One study from India has also reported a similar gender difference in the incidence of Osteogenic sarcoma [9,10]. Nevertheless, female preponderance that had been shown in soft tissue sarcoma could be related to the significant number of cases of Leiomyosarcoma arising from the uterine muscle.

Successful management of sarcomas and localised Osteogenic sarcomas requires careful coordination and timing of staging studies, biopsy, surgery, and preoperative and postoperative chemotherapy. Majority of the cases of localised Osteogenic sarcomas did receive neoadjuvant chemotherapy in our study before proceeding with definitive surgery. It was in keeping with the revised standards for the management of Osteogenic sarcoma that had been established in 1990 [2]. Limb-salvage surgery has now been considered a standard operation for selected cases. Approximately 95% of Osteogenic sarcomas can be treated successfully with this technique [2]. Many studies have targeted the quality of life issues with limb salvage versus amputation but with conflicting results [11,12].

Despite multidisciplinary involvement and appropriate planning of individual cases, the recurrence rate could not be compared between limb salvage and amputation; and also between two groups of limb salvage surgeries who relapse versus who do not relapse mainly because of smaller sample size. However, the recurrence free survival for Osteogenic sarcoma was comparable to few studies as per reported literature but definitely inferior for soft tissue sarcoma [13]. Therefore, we need larger studies and longer follow up period before commenting on this any further.

The two most important determinant of local recurrence in previous studies were the surgical margins and the response to chemotherapy and our study corroborated these findings in emphasizing the importance of negative surgical margins for successful management of sarcoma [13]. In our cohort, 3 patients had positive margins after limb salvage surgery. One of them is alive without recurrence but this patient had 90 percent necrosis on surgical specimen. One had local recurrence in 6 months time which had been treated with re-excision and the third one had multiple recurrences treated with surgery and chemotherapy and subsequently lost to follow. This patient had poor response to neo-adjuvant chemotherapy with significant residual tumour initially. This again highlights the importance of response to chemotherapy for recurrence free survival. The rest of the patients who relapsed had distant recurrences rather than local.

The soft tissue sarcoma group was, somewhat, under represented in our study mainly due to variable pattern of referrals i.e. most patients with low grade soft tissue sarcoma do not follow the oncologists after primary surgical resection. We primarily see patients with high grade tumours referred for adjuvant chemotherapy or for palliative chemotherapy in un-resectable cases. Adjuvant chemotherapy for high grade soft tissue sarcoma remains divisive but has been given to most patients with high grade tumours based on results of earlier meta-analyses which have demonstrated a reduction in local and distant recurrence rate and trend towards improved overall survival with adjuvant chemotherapy [14,15]. Recent trials have also shown an advantage in disease free survival and overall survival with Ifosfamide and Adriamycin combination chemotherapy [16,17]. Radiotherapy has also been used for improving the local control both before and after surgery depending on the individual case of high grade and large soft tissue sarcoma [18].

A major limitation of the study was the wide variation in treatment policies in small number of patients and hence, the analysis was based on small groups of heterogeneous sarcoma population. Also, the lost to follow rate was significantly high in our study for many reasons. The financial burden of the treatment is substantial, and people tend to disappear once the treatment is declared completed for the fear of more expenses associated with regular follow up visits. For the same reason significant number of patients have the tendency not to complete their treatment as soon as they start feeling better and then return later with extensive disease. Yet another factor is inadequate literacy level due to which many fail to understand the importance of follow up visits. Some people even argue about the significance of these visits once the treatment is deemed completed.

## Conclusion

Sarcoma, though a rare cancer group, is associated with considerable morbidity and disability in younger group of the community. Our results showed the different characteristics of sarcoma patients, their course and outcome over a four year period. Multidisciplinary involvement is essential for appropriate and successful management of individual cases. The median recurrence free survival was comparable in our study to the reported literature but with significant lost to follow rate. Further large scale, multicentre prospective studies are needed to have a more comprehensive understanding of the behaviour and outcome of this heterogeneous disease in our population. Also, there is a need for increasing awareness among general public for meticulous follow up.

## Competing interests

The authors declare that they have no competing interests.

## Authors' contributions

TZA and NM conceived the study. TZA performed the literature review, designed the study, formulated the questionnaire, carried out the statistical analysis and wrote the main manuscript. NM supervised the study and proofread the manuscript.

AP and RZJ collected the data by reviewing the files, filled in the questionnaires, entered the data in SPSS and helped in the analysis. SNN, AAZ, and MU contributed in the study subjects from their patients' pool. All authors read and approved the final manuscript.

## References

[B1] Mazanet R Antman Karen H Sarcoma of Soft Tissue and Bone Cancer 1991 68 463 473 10.1002/1097-0142(19910801)68:3<463::AID-CNCR2820680304>3.0.CO;2-E 2065265

[B2] Brennan M Singer S Maki R O'Sullivan B DeVita VT, Hellman S, Rosenberg SA Sarcomas of the soft tissues and bone Cancer: Principles and Practice of Oncology 2008 8 1742 1833

[B3] Coindre JM Terrier P Guillou L Le Doussal V Collin F Ranchere D Sastre X Vilain MO Bonichon F N'guyen BB Predictive value of grade for metastasis development in the main histologic types of adult soft tissue sarcomas: A study of 1240 patients from the French Federation of Cancer Centers Sarcoma Group Cancer 91 2001 10 1914 26 10.1002/1097-0142(20010515)91:10<1914::aid-cncr1214>3.0.co;2-3 11346874

[B4] Vraa S Keller J Nielsen OS Sneppen O Jurik AG Jensen OM Prognostic factors in soft tissue sarcomas: the Aarhus experience Eur J Cancer 34 1998 12 1876 82 10.1016/s0959-8049(98)00233-0 10023309

[B5] Adult soft tissue sarcoma treatment http://cancer.gov/cancertopics/pdq/treatment/adult-soft-tissue-sarcoma/healthprofessional#Section_229

[B6] Grier HE Krailo MD Tarbell NJ Link MP Fryer CJH Pritchard DJ Gebhardt MC Dickman PS Perlman EJ Meyers PA Donaldson SS Moore S Rausen AR Vietti TJ Miser JS Addition of ifosfamide and etoposide to standard chemotherapy for Ewing's sarcoma and primitive neuroectodermal tumor of bone N Engl J Med 2003 348 8 694 701 10.1056/NEJMoa020890 12594313

[B7] Link MP Goorin AM Miser AW Green AA Pratt CB Belasco JB Pritchard J Malpas JS Baker AR Kirkpatrick JA Ayala AG Shuster JJ Abelson HT Simone JV Vietti TJ The effect of adjuvant chemotherapy on relapse-free survival in patients with osteosarcoma of the extremity New Eng J Med 1986 314 25 1600 1606 10.1056/NEJM198606193142502 3520317

[B8] Damron TA Ward WG Stewart A Osteosarcoma, chondrosarcoma, and Ewing's sarcoma: National Cancer Data Base Report Clin Orthop Relat Res 2007 459 40 7 17414166 10.1097/BLO.0b013e318059b8c9

[B9] Yeole BB Jussawalla DJ Descriptive epidemiology of bone cancer in greater Bombay Indian J Cancer 1998 35 101 6 10226399

[B10] Guo W Xu W Huvos AG Healey JH Feng C Comparative frequency of bone sarcomas among different racial groups Chin Med J (Engl) 1999 112 1101 4 11721448

[B11] Greenberg DB Goorin A Gebhardt MC Gupta L Stier N Harmon D Mankin H Quality of life in osteosarcoma survivors Oncology (Huntingt) 1994 8 11 19 discussion 25, 32, 35 7826837

[B12] Christ GH Lane JM Marcove R Psychosocial adaptation of long term survivors of bone sarcoma J Psychosocial Oncol 1995 13 1 22

[B13] Bacci G Ferrari S Mercuri M Bertoni F Picci P Manfrini M Gasbarrini A Forni C Cesari M Campanacci M Predictive factors for local recurrence in osteosarcoma: 540 patients with extremity tumors followed for minimum 2.5 years after neoadjuvant chemotherapy Acta Orthop Scand 1998 69 3 230 10.3109/17453679809000921 9703394

[B14] Tierney JF Stewart LA Parmar MKB Sarcoma Meta-analysis Collaboration Adjuvant chemotherapy for localised resectable soft-tissue sarcoma of adults: meta-analysis of individual data Lancet 1997 350 9092 1647 54 9400508

[B15] Frustaci S Gherlinzoni F De Paoli A Bonetti M Azzarelli A Comandone A Olmi P Buonadonna A Pignatti G Barbieri E Apice G Zmerly H Serraino D Picci P Adjuvant chemotherapy for adult soft tissue sarcomas of the extremities and girdles: results of the Italian randomized cooperative trial J Clin Oncol 2001 19 5 1238 47 11230464 10.1200/JCO.2001.19.5.1238

[B16] Woll PJ Van Glabbeke M Hohenberger P Le Cesne A Gronchi A Hoekstra HJ Radford JA Van Coevorden F Blay J EORTC Soft Tissue & Bone Sarcoma Group Adjuvant chemotherapy with doxorubicin and ifosfamide in resected soft tissue sarcoma: Interim analysis of randomised phase III trial J Clin Oncol 2007 25 18S 10008

[B17] Pervaiz N Colterjohn N Farrokhyar F Tozer R Figueredo A Ghert M A systematic meta-analysis of randomized controlled trials of adjuvant chemotherapy for localised resectable soft-tissue sarcoma Cancer 2008 113 3 573 81 10.1002/cncr.23592 18521899

[B18] Yang JC Chang AE Baker AR Sindelar WF Danforth DN Topalian SL DeLaney T Glatstein E Steinberg SM Merino MJ Rosenberg SA Randomized prospective study of the benefit of adjuvant radiation therapy in the treatment of soft tissue sarcomas of the extremity J Clin Oncol 1998 16 1 197 203 9440743 10.1200/JCO.1998.16.1.197

